# Combined Approach to Stroke Thrombectomy Using a Novel Short Flexible Aspiration Catheter with a Stent Retriever: Preliminary Clinical Experience

**DOI:** 10.1007/s00062-021-01065-7

**Published:** 2021-07-20

**Authors:** Sebastian Remollo, Mikel Terceño, Mariano Werner, Carlos Castaño, María Hernández-Pérez, Jordi Blasco, Luis San Román, Pepus Daunis-i-Estadella, Santiago Thió-Henestrosa, Víctor Cuba, Alfredo Gimeno, Josep Puig

**Affiliations:** 1 Department of Neurosciences, Hospital Universitari Germans Trias i Pujol, Interventional Neuroradiology Unit, Carretera del Canyet, s/n, 08946 Badalona, Spain; 2 Stroke Unit, Hospital Universitari Dr. Josep Trueta, Girona, Spain; 3 Stroke Unit, Department of Neurosciences, Hospital Universitari Germans Trias i Pujol, Badalona, Spain; 4 Neurointerventional Department C.D.I, Hospital Clinic of Barcelona, Barcelona, Spain; 5 Department of Computer Science, Applied Mathematics and Statistics, University of Girona, Girona, Spain; 6 Interventional Neuroradiology, Hospital Universitari de Bellvitge, L’Hospitalet de Llobregat, Spain; 7 Radiology Department, University Hospital of Vall d’Hebron, Barcelona, Spain; 8 IDI-Radiology, University Hospital Dr Josep Trueta, Girona, Spain

**Keywords:** Brain ischemia, Stroke, Mechanical thrombectomy, Device, Combined approach thrombectomy

## Abstract

**Background:**

Large-bore aspiration catheters enabling greater flow rates and suction force for mechanical thrombectomy might improve outcomes in patients with stroke secondary to large-vessel occlusion. Complete or near-complete reperfusion after a single thrombectomy pass (first-pass effect) is associated with improved clinical outcomes. We assessed the efficacy and safety of novel MIVI Q™ aspiration catheters in combination with stent-retriever devices.

**Methods:**

We retrospectively analyzed demographics, procedure characteristics, and clinical data from consecutive patients with acute anterior large-vessel occlusion treated with a combined approach using MIVI Q™ aspiration catheters and stent retrievers. Reperfusion was defined according to the modified thrombolysis in cerebral infarction (mTICI) score. Clinical outcomes were measured by the National Institute of Health Stroke Scale (NIHSS) and modified Rankin scale (mRS) scores.

**Results:**

We included 52 patients (median age, 75 y IQR: 64–83); 31 (59.6%) women; 14 (26.9%) with terminal internal carotid artery occlusions, 26 (50%) middle cerebral artery (MCA) segment M1 occlusions, and 12 (23.1%) MCA segment M2 occlusions; median NIHSS score at admission was 19 (IQR: 13–22). After the first pass, 25 (48%) patients had mTICI ≥ 2c. At the end of the procedure, 47 (90.4%) had mTICI ≥ 2b and 35 (67.3%) had mTICI ≥ 2c. No serious device-related adverse events were observed. Symptomatic intracranial hemorrhage developed in 1 patient. Mean NIHSS score was 13 at 24 h and 5 at discharge. At 90 days, 24 (46.2%) patients were functionally independent (mRS 0–2).

**Conclusion:**

This preliminary study found good efficacy and safety for MIVI Q™ aspiration catheters used in combination with stent-retriever devices.

**Supplementary Information:**

The online version of this article (10.1007/s00062-021-01065-7) contains supplementary material, which is available to authorized users.

## Introduction

Randomized clinical trials have demonstrated the efficacy of mechanical thrombectomy for acute ischemic stroke secondary to large-vessel occlusion [1–5]. Mechanical thrombectomy aims to achieve complete reperfusion, ideally after a single pass of the thrombectomy device through the occluded segment (termed the first-pass effect, FPE) [6, 7]. The FPE is an independent predictor of good clinical outcome [8], and the first-pass rate is a metric of technical success. Modern mechanical thrombectomy devices are associated with higher reperfusion rates, shorter procedure times, better clinical outcomes, and lower risk of mortality [8]; however, although the physical properties influencing the effectiveness of different large-bore aspiration catheters have been investigated [9], the superiority of any specific device over another has yet to be demonstrated in clinical trials [10].

The MIVI Q^TM^ (MIVI Neuroscience, Eden Prairie, MN, USA) is a novel CE-approved device designed to provide greater suction and flow [11]. We assessed the technical efficacy and safety of this new catheter when used in combination with stent-retriever devices in patients with acute stroke due to anterior circulation large-vessel occlusion.

## Methods

### Patients

We retrospectively studied consecutive patients with acute ischemic stroke due to large-vessel occlusion in the anterior circulation (terminal internal carotid artery or middle cerebral artery (MCA) segments M1 or M2, excluding those with simultaneous cervical carotid and intracranial tandem occlusions) who were treated with MIVI Q^TM^ aspiration catheters in combination with stent retrievers as the first-line treatment in a comprehensive stroke center between June 2019 and May 2020. All thrombectomy procedures were performed by 4 interventional neuroradiologists, all of whom had ≥ 4 years experience in endovascular stroke treatment. The decision to use the MIVI Q^TM^ aspiration catheters as well as the type of stent retriever was at the discretion of the operator. Inclusion criteria were age ≥ 18 years, absence of pregnancy, acute ischemic stroke from large-vessel occlusion confirmed by computed tomography angiography or magnetic resonance angiography, premorbid modified Rankin scale (mRS) score ≤ 2, clinically significant neurological deficit, defined as assessed National Institutes of Health Stroke Scale (NIHSS) score ≥ 6 and time from last seen well to treatment ≤ 24 h. Intravenous thrombolysis was administered before mechanical thrombectomy in all eligible patients according to established criteria [12]. The study received institutional review board approval. After mechanical thrombectomy, patients or their representatives provided informed consent for imaging, procedural, analytical, and clinical data to be used in future retrospective studies. Our institution’s ethics committee waived the need for further specific informed consent for the current study.

### Data Collection

We prospectively recorded age, sex, comorbidities, NIHSS score at admission and 24 h after mechanical thrombectomy, Alberta stroke program early CT score (ASPECTS) at admission, site of occlusion, timing of medical and interventional treatments, devices used, reperfusion outcomes, intracranial hemorrhage (ICH) and symptomatic ICH (sICH) [13] on 24‑h follow-up imaging, device-related or technique-related adverse events, and modified Rankin scale (mRS) score at day 90.

### Outcome Variables

We recorded puncture-to-revascularization time, divided into puncture-to-first-run time and first-run-to-final-revascularization time. To determine procedural efficacy, an independent experienced interventional neuroradiologist blinded to the interventional team’s assessment classified technical success after a single pass according to the modified thrombolysis in cerebral infarction (mTICI) scale as FPE (mTICI ≥ 2c) or true FPE (tFPE = mTICI 3) [14, 15], as well as final reperfusion scores according to the same criteria.

To determine procedural safety, we analyzed ICH according to the European Cooperative Acute Stroke Study-II definition [13] on computed tomography 24 h after the procedure, early neurological deterioration (≥ 4 points from baseline NIHSS 24 h after the procedure), clinical complications deemed procedural by the attending interventionist, and deaths within 30 days attributable to the procedure. Clinical outcomes were assessed by the mRS score at 90 days. Good clinical outcome was defined as mRS ≤ 2.

### Thrombectomy Technique

The MIVI Q^TM^ is a novel aspiration catheter designed to maximize flow and minimize pressure loss [11]. The proximal three quarters of the catheter shaft have been replaced with a 119 cm 0.020″ stainless steel pusher wire. This innovation allows the full internal area of the guiding catheter to provide a higher aspirated flow rate and suction force than standard tubular catheter designs. The outer diameter (OD) of the proximal segments of the Q^TM^ is 0.088″; the length of the catheter and the inner diameter (ID) of the distal catheter segments varies according to the model, being 25 cm and 0.069″ for the Q6, 25 cm and 0.057″ for the Q5, 30 cm and 0.043″ for the Q4, and 43 cm and 0.036″ for the Q3. The aspiration catheter is used with the Super 90 8F guide catheter (MIVI Neuroscience Inc.) (ID 0.090″, length 80 cm, 90 cm, or 95 cm). Pump aspiration is directly applied to the Super 90 guide catheter and the total system length varies as the Q^TM^ is extended outward and retracted into the Super 90 guide catheter [11].

**Fig. 1:**
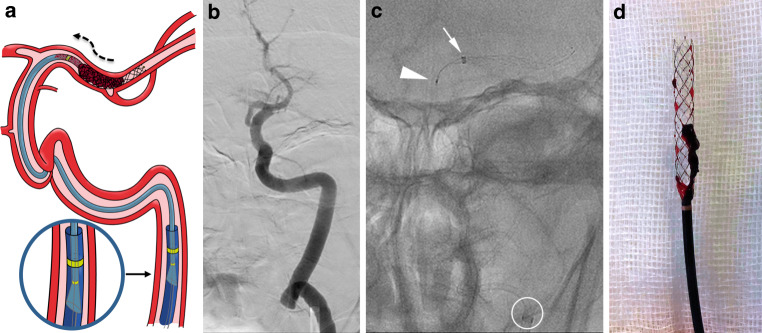
**a** Illustration of the Q^TM^ and stent retriever combined approach during the thrombus wedging maneuver. After removing the microcatheter, the stent retriever has been slightly retracted and the Q^TM^ catheter carefully advanced under pump aspiration to ensure better engagement of the thrombus. The flared proximal tip of the Q catheter (*magnified inset*) ensures a tight seal between its outer surface and the Super 90 guiding catheter’s inner surface, enabling increased suction power (*curved black dashed arrow*) directly through the guide catheter. **b** Anteroposterior left internal carotid artery (ICA) angiography through the Super 90 guiding catheter showing a terminal ICA occlusion. **c** Unsubtracted anteroposterior view showing the combined approach thrombectomy: Distal end of the Super 90 Catheter (*white circle*), distal end of a Q6 aspiration catheter (*white arrow*), and a fully deployed Aperio® Hybrid stent with its proximal end inside the Q6 (*white arrowhead*). **d** Q6 and Aperio® Hybrid with the “pinned” thrombus

Depending on the patient’s condition, procedures were done with the patient under local anesthesia, conscious sedation or general anesthesia. Through femoral artery access, the guide catheter was placed in the internal carotid artery of the affected side. A Rebar^TM^18 microcatheter (ev3 Inc. Medtronic, Minneapolis, MN, USA) with a Synchro® 0.014″ microwire (Boston Scientific for Stryker Neurovascular, Fremont, CA, USA) was advanced through the arterial occlusion. The largest diameter Q^TM^ aspiration catheter that would fit in the affected vessel was advanced to the occlusion over the microcatheter and microwire unit in a monorail fashion. Then a stent retriever was advanced through the microcatheter and deployed at the level of the thrombus using the push-and-fluff technique [16]. To increase the cross-sectional area and flow, the microcatheter was removed [17] and pump aspiration was applied to the guiding catheter. The stent retriever was retracted slightly and the Q^TM^ was carefully advanced until no flow was present in the aspiration tubing. After 1 min under occlusive aspiration, both devices were slowly retrieved as a unit inside the guiding catheter and then withdrawn while maintaining aspiration (Fig. 1).

## Results

During the 10-month study period, a total of 105 patients with acute ischemic stroke underwent mechanical thrombectomy at Hospital Universitari Germans Trias i Pujol comprehensive stroke center. The MIVI Q^TM^ aspiration catheter was the first-line treatment in 74 patients with large-vessel occlusion. Of these, 22 were excluded from the analyses (13 had posterior circulation occlusions, 4 had tandem occlusions in the cervical internal carotid and MCA, and 5 had anterior circulation stroke but were treated with first-line contact aspiration only). Thus, 52 patients were analyzed. A flowchart of patient selection is provided in Supplemental Fig. 2. Table 1 summarizes the demographic, clinical, and procedure characteristics of our cohort.

**Table 1:** Baseline characteristics of the 52 patients included in the study

Characteristics	Values
*Age (years) median (IQR)*	75 (64–83)
*Female sex, n (%)*	31 (59.6)
*NIHSS score at admission, median (IQR)*	19 (13–22)
*Pre-stroke mRS, n (%)*	
0	44 (84.6)
1	4 (7.7)
2	4 (7.7)
*Medical history, n (%)*	
Hypertension	34 (65.4)
Diabetes mellitus	15 (28.8)
Atrial fibrillation	13 (25)
Dyslipidemia	28 (53.8)
Smoking	12 (23.1)
Obesity	5 (9.6)
Myocardial infarction or coronary artery disease	13 (25)
Previous stroke or TIA	8 (15.4)
Anticoagulation	13 (25)
Antiplatelet	9 (17.3)
*ASPECTS at admission, median (IQR)*	9 (8–10)
*Occlusion site, n (%)*	
Left side	30 (57.7)
ICA‑T	14 (26.9)
MCA-M1	26 (50)
MCA-M2	12 (23.1)
*Time from symptom onset or LTSW to arterial puncture (min), median (IQR)*	278 (170–419)
*Door-to-groin time (min), median (IQR)*	70 (36–96)
*Q*^*TM*^* device used, n (%)*	
Q3	5 (9.6)
Q4	7 (13.5)
Q5	19 (36.5)
Q6	21 (40.4)
*Stent retriever used, n (%)*	
Aperio Hybrid®	27 (51.9)
Catch View®	15 (28.8)
Embotrap II®	5 (9.6)
NeVa®	5 (9.6)
*IV tPA administered, n (%)*	17 (32.7)

*IQR* interquartile range, *NIHSS* National Institute of Health Stroke Scale score, *mRS* modified Rankin Scale score, *TIA* transient ischemic attack, *ASPECTS* Alberta Stroke Program Early CT Score, *MCA-M1* ICA-T: intracranial carotid T occlusion, M1 segment of the middle cerebral artery, *MCA-M2* M2 segment of the middle cerebral artery, *LTSW* last time seen well, Q^TM^ (MIVI Neuroscience, Inc., Eden Prairie, MN, USA), Aperio Hybrid® (Acandis company, Pforzheim, Germany), Catch View® (Balt, Montmorency, France), Embotrap II® (Cerenovus, Galway, Ireland), NeVa® (Vesalio, Nashville, TN, USA), *IV tPA* intravenous tissue plasminogen activator

The Q^TM^ aspiration catheter reached the occlusion in all patients, achieving FPE in 48%. Final reperfusion was excellent (mTICI ≥ 2c) in 67.3% and successful (mTICI ≥ 2b) in 90.4%. No device-related adverse events occurred, and no patients required rescue therapy. Table 2 reports procedural variables.

**Table 2:** Angiographic outcomes

Reperfusion and time-metrics	Results
*Groin-puncture-to-revascularization (min), median (IQR)*	42 (22–66)
*Time from first angiogram to recanalization (min), median (IQR)*	28 (15–59)
*Q*^*TM*^* tFPE*	
mTICI 3, *n* (%)	22 (42.3)
Groin-puncture-to-revascularization (min), median (IQR)	29 (20–45)
Time from first angiogram to recanalization (min), median (IQR)	15 (11–26)
*Q*^*TM*^* FPE*	
mTICI 2c‑3, *n* (%)	25 (48)
Groin-puncture-to-revascularization (min), median (IQR)	26 (20–39)
Time from first angiogram to recanalization (min), median (IQR)	15 (11–24)
*Q*^*TM*^* mTICI* *≥* *2b with a single pass*	
mTICI ≥ 2b with a single pass, *n* (%)	28 (53.8)
Groin-puncture-to-revascularization (min), median (IQR)	26 (20–41)
Time from first angiogram to recanalization (min), median (IQR)	15 (11–26)
*≤* *2Q*^*TM*^* passes*	
mTICI ≥ 2b, *n* (%)	34 (65.4)
mTICI 2c‑3, *n* (%)	29 (55.7)
Total passes, *n* (mean)	40 (1.18)
Groin-puncture-to-revascularization (min), median (IQR)	26 (20–46)
Time from first angiogram to recanalization (min), median (IQR)	15 (11–34)
*≤* *3Q*^*TM*^* passes*	
mTICI ≥ 2b, *n* (%)	39 (75)
mTICI 2c‑3, *n* (%)	32 (61.5)
Total passes, *n* (mean)	55 (1.41)
Groin-puncture-to-revascularization (min), median (IQR)	32 (20–55)
Time from first angiogram to recanalization (min), median (IQR)	18 (14–40)
*Final Reperfusion*	
mTICI 3, *n* (%)	28 (53.8)
mTICI ≥ 2b, *n* (%)	47 (90.4)
mTICI 2c‑3, *n* (%)	35 (67.3)
Total passes, mean	2.46

*IQR* interquartile range, *mTICI* modified thrombolysis in cerebral ischemia, *tFPE* mTICI = 3 after a single pass, FPE mTICI ≥ 2c after a single pass

Table 3 reports the clinical outcomes related to safety and efficacy. In one patient with an M1 occlusion, an embolus that had migrated into the A2 segment of the anterior cerebral artery (unaffected territory) during withdrawal was retrieved uneventfully with the same system. A patient in his 40s (admission ASPECTS 5) treated with intravenous thrombolysis who achieved mTICI 2b after 5 passes developed sICH and died. In another patient, microwire vessel perforation during microcatheter advancement (unrelated to the aspiration catheter or the stent retriever) resulted in a parenchymal hematoma (PH1), identified at 24h follow-up, but did not cause any neurological deterioration. Petechial reperfusion hemorrhages (HI1) or minor subarachnoid hemorrhage occurred in 8 patients.

**Table 3:** Clinical efficacy and safety outcomes

Efficacy and safety	Results
*24-hour NIHSS, mean (IQR)*	13 (4–18)
*Early neurological improvement, n (%)*^a^	28 (53.8)
*NIHSS at discharge, mean (IQR)*	5 (1–11)
*mRS at discharge, n (%)*	
0–1	8 (15.4)
0–2	15 (28.8)
3	8 (15.4)
4	13 (25)
5	7 (13.5)
6	9 (17.3)
*mRS at 90 days, n (%)*	
0–1	20 (38.5)
0–2	24 (46.2)
3	4 (7.7)
4	6 (11.5)
5	3 (5.8)
6	15 (28.8)
*Intracranial hemorrhage, n (%)*	
sICH	1 (1.9)
All ICH	10 (19.2)
*Early neurological deterioration, n (%)*	7 (13.5)
*All-cause mortality at 90 days, n (%)*	15 (28.8)
*In hospital mortality, n (%)*	9 (17.3)
*Serious device-related adverse events, n (%)*	0 (0)
*Serious procedure related adverse events, n (%)*^b^	1 (1.9)

*NIHSS* National Institute of Health Stroke Scale score, *IQR* interquartile range, *mRS* modified Rankin Scale score, *sICH* symptomatic intracranial hemorrhage, *ICH* intracranial hemorrhage

^a^ Early neurological improvement was defined as a reduction of ≥ 5 points on the NIHSS or an NIHSS score < 4 at 24-hours

^b^ Microwire perforation with contrast extravasation on angiography

Early neurological deterioration developed in 7 (13.5%) patients. A total of 9 (17.3%) patients died before hospital discharge, 2 from malignant edema related to large-volume stroke. At 90 days 24 (46.2%) patients were functionally independent (mRS 0–2).

## Discussion

Speed and grade of recanalization are of paramount importance in neurothrombectomy; therefore, choosing a safe and time-efficient strategy is key in stroke treatment.

Final reperfusion rates and functional outcomes after thromboaspiration and stent-retriever thrombectomy are similar [18]. Used as a stand-alone first-line technique, stent retrievers achieve better reperfusion with less need for rescue devices but require longer groin-to-reperfusion times [18]. Recently introduced approaches combining stent retrievers and large-bore aspiration catheters trap the thrombus between the catheter tip and the stent retriever while maintaining local aspiration [19, 20], a change from earlier combined approaches (e.g., Solumbra) where the stent retriever was retracted into the aspiration catheter [21]. Combined approaches have shown improved reperfusion rates and FPE [22]; however, there remains considerable room for improvement in achieving an early and complete reperfusion [23].

Successful thromboaspiration requires using the largest catheter that the vessel can accommodate [24–26]. The MIVI Q™ aspiration catheter is designed to maximize lumen size, increasing flow rates and tip suction force [11]. Both properties combined have been shown to be necessary to effectively extract the clot, but in vitro experiments indicated that suction force is more relevant than aspiration flow in terms of efficacy, particularly in hard clots [27]. The MIVI Q^TM^ exhibits these two physical properties, and especially the aspiration flow rate benefits from a dynamic effect as in vitro testing has demonstrated that it increases as the Q^TM^ is retracted inside the guiding catheter [11]. It can be used alone or combined with a stent retriever, as in our cohort. In this preliminary clinical experience, there were no device-related adverse events, and the angiographic, time-metric, and clinical outcomes were similar to those in other studies using combined mechanical thrombectomy approaches [20, 28–30].

Delivering large-bore aspiration catheters intracranially to the thrombus is not always easy [31] because of vessel tortuosity, underlying intracranial atherosclerosis, or even the anatomical position where the ophthalmic artery originates from the carotid siphon [32]. The soft and flexible design of the MIVI Q™ aspiration catheter resulted in excellent trackability; in all patients, the catheter was navigated to the occlusion uneventfully. These results compare favorably with those reported in an initial experience with the Sofia catheter (MicroVention, Tustin, CA, USA), where the occlusion was reached uneventfully in 96% of cases [33]. The Q™ catheter was coaxially advanced over the Rebar^TM^18 microcatheter, and no stent anchoring [34] or “blind exchange” maneuvers [35] were needed to navigate the Q™ catheter, even when using larger caliber catheters and when the arterial anatomy was tortuous. Avoiding anchoring and exchange maneuver eliminates a theoretical risk of vascular injury.

The Q™ catheter design takes advantage of fluid mechanics theory so that the suction force in the tip of the Q™ catheter increases when it is retrieved inside the guiding catheter, potentially diminishing the risk of distal emboli migrating during system retrieval [11]. This may explain the low rate of complications related to emboli migrating to previously unaffected territories (1.9%) in our series.

Another potential advantage of the MIVI Q™ aspiration catheter design is that it does not require a rotating hemostatic valve connected to a saline flush line, thus enabling faster preprocedural preparation and faster purging after its retrieval when additional passes are necessary and thereby improving the efficiency of interventions.

Our successful reperfusion rate (90.4%) is in line with those reported by Hesse et al. [30] in the primary combined approach (PCA) group (86%), CAPTIVE (100%) [20], and SAVE (95%) [28]. Likewise, our rate of final TICI 3 (53.8%) is in line with those reported in PCA (37.5%), CAPTIVE (33%), and SAVE (56%). The proportion of procedures that achieved mTICI ≥ 2b with a single pass (53.8%) is in line with rates reported in CAPTIVE (59%) and SAVE (74%). Our rate of tFPE (42.3%) is similar to that reported in SAVE (45%), but higher than that reported by Hesse et al. [30] (26%), who used the Solumbra technique [21] for most cases in the PCA group.

Median groin-to-recanalization time was 42 min, similar to that reported in SAVE (34 min) [28] and PCA (51 min) [30], but longer than in CAPTIVE (14 min) [20].

Lastly, 46.2% of patients in our cohort had good clinical outcomes (mRS ≤ 2) at 90-days, comparable to the 49% reported in CAPTIVE [20].

In summary, the procedure times and proportion of patients achieving FPE, tFPE, excellent and successful final reperfusion and good clinical outcomes at 3 months, were in line with those reported in other cohorts where combined approaches were used.

### Limitations

This retrospective, non-randomized study included relatively few patients at a single center with no control group, and reperfusion and clinical outcomes were local operator-measured; thus, caution is essential in extrapolating the results. Nevertheless, our preliminary results include the learning curve inherent in using new devices and merit further studies with larger samples to determine the efficacy of this approach compared to other devices and approaches.

## Conclusion

This preliminary study suggests that the MIVI Q^TM^ aspiration catheter has a good efficacy and safety profile when used in combination with stent retrievers, achieving high reperfusion rates and favorable clinical outcomes.

## Supplementary Information

**Supplemental Fig. 2.** Flowchart of subjects with acute ischemic stroke caused by a large-vessel occlusion selected for analysis. *LVO* large-vessel occlusion, *MT* mechanical thrombectomy, *SR* stent-retriever

## Abbreviations

ASPECTS: Alberta stroke program early CT score
CAPTIVE: Continuous aspiration prior to intracranial vascular embolectomy
CT: Computed tomography
FPE: First-pass effect
ICH: Intracranial hemorrhage
IQR: Interquartile range
LTSW: Last time seen well
LVO: Large-vessel occlusion
MCA: Middle cerebral artery
MRS: Modified Rankin scale
MTICI: Modified thrombolysis in cerebral infarction scale
NIHSS: National Institutes of Health Stroke Scale
PCA: Primary combined approach
SAVE: Stent-retriever assisted vacuum-locked extraction
SICH: Symptomatic intracranial hemorrhage
TFPE: True first-pass effect
TIA: Transient ischemic attack
TPA: Tissue plasminogen activator
