# Supplementary material for: Combined Approach to Stroke Thrombectomy Using a Novel Short Flexible Aspiration Catheter with a Stent Retriever: Preliminary Clinical Experience
Source: Clin Neuroradiol. 2021 Jul 20;32(2):393–400. doi: 10.1007/s00062-021-01065-7 (PMC9187553; doi:10.1007/s00062-021-01065-7)
Supplement: Supplementary file 1 — Supplemental Fig. 2. Flowchart of subjects with acute ischemic stroke caused by a large-vessel occlusion selected for analysis. LVO large-vessel occlusion, MT mechanical thrombectomy, SR stent-retriever [file 62_2021_1065_MOESM1_ESM.pdf]

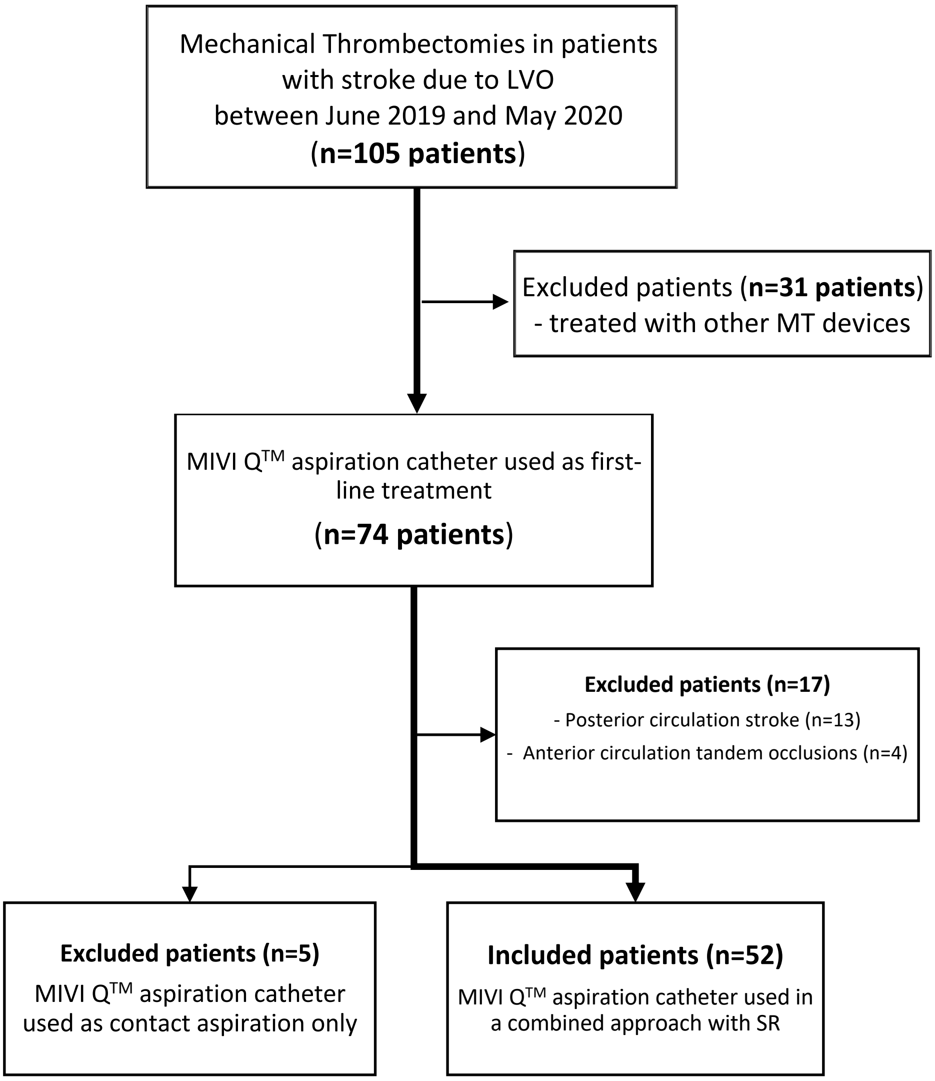

Supplemental Figure 2. Flowchart of subjects with acute ischemic stroke caused by a large-vessel occlusion selected for analysis. LVO: large-vessel occlusion, MT: Mechanical Thrombectomy, SR: Stent-Retriever
